# The Effects of Neuropeptide Y Overexpression on the Mouse Model of Doxorubicin-Induced Cardiotoxicity

**DOI:** 10.1007/s12012-019-09557-2

**Published:** 2019-12-06

**Authors:** Minttu Mattila, Mirva Söderström, Liisa Ailanen, Eriika Savontaus, Mikko Savontaus

**Affiliations:** 1 Research Centre for Integrative Physiology and Pharmacology, Institute of Biomedicine, University of Turku, Turku, Finland; 2 Drug Research Doctoral Programme, University of Turku, Turku, Finland; 3 Department of Pathology, Turku University Hospital and University of Turku, Turku, Finland; 4 Clinical Pharmacology, Turku University Hospital, Turku, Finland; 5 Heart Centre, Turku University Hospital and University of Turku, Turku, Finland

**Keywords:** Doxorubicin, Neuropeptide Y, Cardiotoxicity model, Mouse model

## Abstract

Doxorubicin is a potent anticancer drug with cardiotoxicity hampering its use. Neuropeptide Y (NPY) is the most abundant neuropeptide in the heart and a co-transmitter of the sympathetic nervous system that plays a role in cardiac diseases. The aim of this work was to study the impact of NPY on doxorubicin-induced cardiotoxicity. Transgenic mice overexpressing NPY in noradrenergic neurons (NPY-OE^DβH^) and wild-type mice were treated with a single dose of doxorubicin. Doxorubicin caused cardiotoxicity in both genotypes as demonstrated by decreased weight gain, tendency to reduced ejection fraction, and changes in the expression of several genes relevant to cardiac pathology. Doxorubicin resulted in a tendency to lower ejection fraction in NPY-OE^DβH^ mice more than in wild-type mice. In addition, gain in the whole body lean mass gain was decreased only in NPY-OE^DβH^ mice, suggesting a more severe impact of doxorubicin in this genotype. The effects of doxorubicin on genes expressed in the heart were similar between NPY-OE^DβH^ and wild-type mice. The results demonstrate that doxorubicin at a relatively low dose caused significant cardiotoxicity. There were differences between NPY-OE^DβH^ and wild-type mice in their responses to doxorubicin that suggest NPY to increase susceptibility to cardiotoxicity. This may point to the therapeutic implications as suggested for NPY system in other cardiovascular diseases.

**Electronic supplementary material:**

The online version of this article (10.1007/s12012-019-09557-2) contains supplementary material, which is available to authorized users.

## Introduction

Doxorubicin (DOX) is a member in the anthracycline family and is one of the most effective anticancer drugs. Its use is severely limited by cardiotoxicity that can lead to cardiomyopathy and heart failure [1]. Among cancer survivors, cancer therapy-related heart disease induced by anthracyclines has been shown to be a major cause of morbidity and mortality [2]. In order to develop ways to treat and prevent the adverse cardiac reactions, the mechanisms contributing to DOX-induced cardiotoxicity need to be defined in detail.

Increased sympathetic nervous system (SNS) activity has been shown to play a role in DOX-induced cardiomyopathy [3, 4], and similar to other forms of heart failure, β-blockers significantly improve cardiac function in DOX-induced heart failure [5, 6]. However, sympathetic nerves not only excrete noradrenaline, but also other neurotransmitters that could play a role in the pathogenesis of DOX-induced cardiomyopathy. Neuropeptide Y (NPY) is a co-transmitter in sympathetic nerves, and is the most abundant neuropeptide in the heart [7, 8]. It exerts its effects via Gi-protein-coupled Y-receptors, of which Y1-, Y2- and Y5-receptors are expressed in the heart. The demonstrated actions of NPY in the heart are extensive and affect the contractility of ventricular cardiomyocytes and the excitation–contraction coupling as well as cellular growth, blood supply, and neuronal control [9, 10]. There is evidence linking NPY to different types of cardiovascular diseases with differential, even opposing, effects via diverse mechanisms [8, 9, 11]. We hypothesized that it may also play a role in DOX-induced cardiotoxicity.

Disturbed calcium cycling plays a major role in the pathogenesis of DOX-induced cardiomyopathy [12]. DOX alters deleteriously the expression of many genes specific for cardiac calcium handling including ryanodine receptor (*RyR2*), sarcoplasmic reticulum Ca^2+^ ATPase (*Serca2a*), and phospholamban (*Pln*), a SERCA2a inhibitor [12–14]. DOX leads to decreased *Serca2a* expression thus inhibiting SERCA2a pump, decreases *RyR2* expression, and induces inappropriate opening of the ryanodine receptors [12, 15, 16]. In the failing heart, a decrease in SERCA2a expression and activity results in myocardial dysfunction due to diminished calcium uptake and release by sarcoplasmic reticulum [17]. Y-receptor activation inhibits adenylate cyclase and decreases cAMP/PKA stimulation of L-type Ca^2+^ currents. On the other hand, Y1-receptor has been shown to couple also to Gq protein to modulate calcium transients [18] and increase intracellular Ca^2+^ level [19, 20] in cardiomyocytes. Thus, NPY could have an impact on the disturbed calcium handling induced by DOX.

DOX alters cardiac function also via other mechanisms than calcium handling to induce contractile dysfunction and pathological remodeling. It has been shown to upregulate matrix metalloproteinase 2 (*Mmp2*) [16, 21] which along with matrix metalloproteinase 9 (*Mmp9*) participates in the degradation of sarcomeric and cytoskeletal proteins [12, 22]. DOX has also been reported to increase the β-myosin heavy chain (*Mhc*-*β*) levels [23–25], which together with changes in α-myosin heavy chain (*Mhc*-*α*) associates to altered contractile performance in different cardiomyopathies [24–26]. Moreover, DOX-induced cardiotoxicity involves the inflammatory responses and oxidative stress as evidenced by changes in cytokines, mitochondrial gene, and protein expression [12, 27]. NPY has been linked to the remodeling of myocardial tissue that could be beneficial in short-term, but can lead to cardiac hypertrophy and pathological remodeling [8] in the long run. The demonstrated effects of NPY include reduced degradation and stimulated synthesis of proteins [28, 29], increased survival of myocytes and decreased fibrosis [30] or activation of fibroblasts [31]. NPY may have proinflammatory effects in atherosclerosis [32], but the inflammatory effects have not been studied in the context of heart diseases. However, studies on the effects of NPY on cardiomyocyte mitochondria have demonstrated impaired mitochondrial function and energy metabolism with changes in the levels of PGC-1α, a key regulator of mitochondrial biogenesis [33, 34]. Thus, NPY could impact DOX-induced cardiotoxicity via various mechanisms.

In order to address the question whether NPY has an impact on DOX-induced cardiotoxicity, we treated mice overexpressing NPY in noradrenergic neurons (NPY-OE^DβH^) with DOX and studied the effects on body composition, cardiac structure, and function as well as explored the potential mechanisms. The NPY-OE^DβH^ mouse model was previously generated and was verified to have about twofold increased *Npy* expression in noradrenergic neurons including adrenal gland and brain stem [35, 36]. The level of overexpression is relevant in terms of NPY excess in chronic mild stress and gain-of-function polymorphisms of NPY in humans as the NPY-OE^DβH^ model recapitulates findings in these situations [37]. The metabolic phenotype of NPY-OE^DβH^ mouse has been extensively characterized and includes adult-onset obesity, impaired glucose tolerance, and dyslipidemia [35, 36, 38]. The cardiovascular phenotype has not been studied in detail, but NPY-OE^DβH^ mice are more sensitive to endothelial damage-induced vascular wall hypertrophy, and neointima formation [39]. The aim of the current study was to use the NPY-OE^DβH^ mouse model to elucidate the effects of excess NPY on DOX-induced cardiotoxicity.

## Methods

### Animals

Adult, 8–10 weeks old male homozygous transgenic OE-NPY^DβH^ from homozygous breeders and wild-type C57BL/6N mice (WT) from WT breeders originating from the same heterozygous breedings maintained on a C57BL/6N inbred background were used. The young age was selected to avoid the full metabolic phenotype of OE-NPY^DβH^. Mice were housed individually in a Ventilated Cage System (Scanbur) at 22 ± 1 °C, 55 ± 5% humidity, and on a 12 h dark/light cycle with free access to mouse chow food and tap water ad libitum. All animal work was done with authorization from the National Animal Experiment Board (ELLA), license number: ESAVI/1256/04.10.07/2015.

### Doxorubicin Administration

Cardiotoxicity was induced by administrating DOX (Caelyx 2 mg/ml, at a dose of 20 mg/kg, Janssen Pharmaceutica NV, Belgium) or PBS to mice (*n* = 7–9/group) as a single intraperitoneal injection. The DOX protocol was based on earlier studies including our recent paper where we used the same DOX administration protocol to induce cardiotoxicity [40–42].

### Body Weight and Composition

In order to follow general well-being, the mice were weighted weekly, and the body composition (fat and lean mass) of the mice was measured at week 0 and week 6 by quantitative nuclear magnetic resonance (NMR) scanning with EchoMRI-700 (Echo Medical Systems, Houston, Texas, USA).

### Echocardiography

Cardiac structure and function was analyzed at week six by echocardiography that was conducted with VisualSonics Vevo 2100 ultrasound system (VisualSonics, Toronto, Canada) equipped with a 30-MHz transducer. Mice were anesthetized by inhalation of 4.5% isoflurane. Anesthesia was maintained by isoflurane gating the heart rate between 400 and 500 beats per minute for imaging the heart. The chests of the mice were shaved from the hairs with a chemical hair remover (Veet; Reckitt Benckiser) and gel (Eco Supergel; Ceracarta, Forlì, Italy) was applied to the chest before the placement of the probe. Left ventricular function, dimensions, and heart rate were measured among other parameters in short-axis view.

### Samples and Histology

Mice were sacrificed at 6 weeks after DOX or saline administration, and heart, lungs, and liver of each mouse was weighed. The tibia was collected and its length was recorded. Hearts were cut in half after which the apex of the heart was frozen in liquid nitrogen immediately after collection, stored at − 70 °C, and used later for RNA extraction, and the base of the heart was fixed for 24 h in 4% paraformaldehyde and subsequently transferred to 70% ethanol and embedded in paraffin. Paraffin Sections (5 μm) were prepared and the sections were collected onto Superfrost plus slides (O. Kindler GmbH, Germany). The paraffin sections were stained with hematoxylin and eosin as well as with Van Gieson’s staining for histological analysis. The sections were scanned using Pannoramic 250F Flash III SlideScanner (3dHistech, Hungary). Myocardial degeneration, inflammation, fibrosis, and nuclear atypia were analyzed and scored by two observers, an experienced pathologist (MSö), and the primary investigator (MM). Histological scoring was carried out blinded as to the other investigator and to the treatment status of the mice. Myocardial degeneration, inflammation, fibrosis, and nuclear atypia were scored on a scale from 0 to 3. In case of discrepancy, investigators evaluated the samples together to gain consensus.

### Quantitative Reverse Transcriptase PCR

RNA was extracted from the frozen heart tissue with RNeasy mini kit including DNase treatment (Qiagen, Germany). RNA was transcribed to cDNA with High Capacity RNA-to-cDNA Kit (Applied Biosystems, USA). Quantitative reverse transcriptase PCR was performed using SYBR Green method with Kapa Sybr Fast qPCR Kit (Kapa Biosystems, Woburn, MA, USA) and Applied Biosystems 7300 Real-Time PCR system. Target gene expression was normalized to *S29* housekeeping gene, and primers were fwd 5′-ATGGGTCACCAGCAGCTCTA-3′ and rev 5′-AGCCTATGTCCTTCGCGTACT-3′. The fold induction was calculated using the comparative ΔCt method and presented as relative transcript levels (2^−ΔΔCt^). Primers were for *Anp* fwd 5′-GCTTCCAGGCCATATTGGAG-3′, rev 5′-GGGGGCATGACCTCATCTT-3′; for *Mhc*-*β* fwd 5′-AGGGTGGCAAAGTCACTGCT-3′, rev 5′-CATCACCTGGTCCTCCTTCA-3′; for *Mmp2* fwd 5′-GATGTCGCCCCTAAAACAGAC-3′, rev 5′-CAGCCATAGAAAGTGTTCAGGT-3′; for *Mmp9* fwd 5′-CTGGACAGCCAGACACTAAAG-3′, rev 5′-CTGGCGGCAAGTCTTCAGAG-3′; for *Npy* fwd 5′-CTCCGCTCTGCGACACTAC-3′, rev 5′-GGAAGGGTCTTCAAGCCTTGT-3′; for *Pln* fwd 5′-CACTGTGACGATCACCGAAG-3′, rev 5′-CAGCATCTCGTTTCGCATTA-3′; for *RyR2* fwd 5′-CAGCATCTCGTTTCGCATTA-3′, rev 5′-GGCTGTGTTCCACCTTCAAT-3′; for *Serca2a* fwd 5′-GAGAACGCTCACACAAAGACC-3′, rev 5′-CTTCTTCAGCCGGCAATTCGTTG-3′; and for *Th* fwd 5′-CCCAAGGGCTTCAGAAGAG-3′, rev 5′-GGGCATCCTCGATGAGACT-3′. Additionally, *α*-*Sma, Beta1R, Bnp, Il*-*1β, Mmp13, Npy1R*, *Npy5R*, *Pgc*-*1α*, *Tgf*-*β, and Tnf*-*α* gene expression were studied (sequences available upon request).

### Statistical Analysis

GraphPad Prism 6 software (La Jolla, USA) was used for statistical analyses. Statistical significance was accepted at the level of *p* < 0.05. Statistical significances were determined with unpaired Student’s *t* test when comparing two groups, or with two-way ANOVA using NPY overexpression and DOX as independent variables. In two-way ANOVA, multiple comparisons were analyzed and corrected with Tukey post hoc test when *p*-value for interaction of genotype and treatment effect was < 0.1. Data are presented as mean of absolute values ± standard error mean (SEM). Quantitative PCR values are presented as means ± SEM in relation to the housekeeping gene S29 mRNA level. Formula 2^−ΔΔCT^ was used to calculate the gene expression relative to the expression level of WT saline mice.

## Results

### Weight and Body Composition

**Fig. 1:**
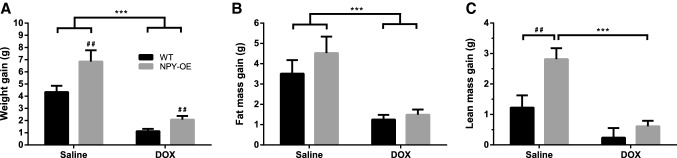
Body weight and composition in saline and doxorubicin (DOX) -treated wild-type (WT) and NPY-OE^DβH^ (NPY-OE) mice (*n* = 7–9/group), **a** body weight gain in 6 weeks, **b** fat mass gain, and **c** lean mass gain. Values are presented as means ± SEM. *** *p* < 0.001 DOX vs. saline, ## *p* < 0.01 NPY vs. WT. **a** Two-way ANOVA genotype and treatment effects, **b** two-way ANOVA treatment effect, **c** Tukey post hoc test following two-way ANOVA

In the beginning of experiment, the weight of the animals (WT saline 24.8 ± 1.0 g, NPY-OE^DβH^ saline 24.2 ± 0.7 g, WT DOX 24.9 ± 0.8 g, NPY-OE^DβH^ DOX 25.0 ± 0.5 g) did not differ between the groups (two-way ANOVA genotype effect *p* = 0.76, treatment effect *p* = 0.52). DOX decreased gain in body weight (Fig. 1a) and fat mass (Fig. 1b), leading to significant difference between saline and DOX-treated mice. Moreover, in the NPY-OE^DβH^ mice, weight gain was significantly increased compared to WT mice in both saline and DOX-treated groups (Fig. 1a), an effect which was not seen in fat mass gain (Fig. 1b). DOX decreased lean mass gain, which was statistically significant only in the NPY-OE^DβH^ mice, which showed higher gain on the control treatment (Fig. 1c).

### Echocardiography Analysis

**Fig. 2:**
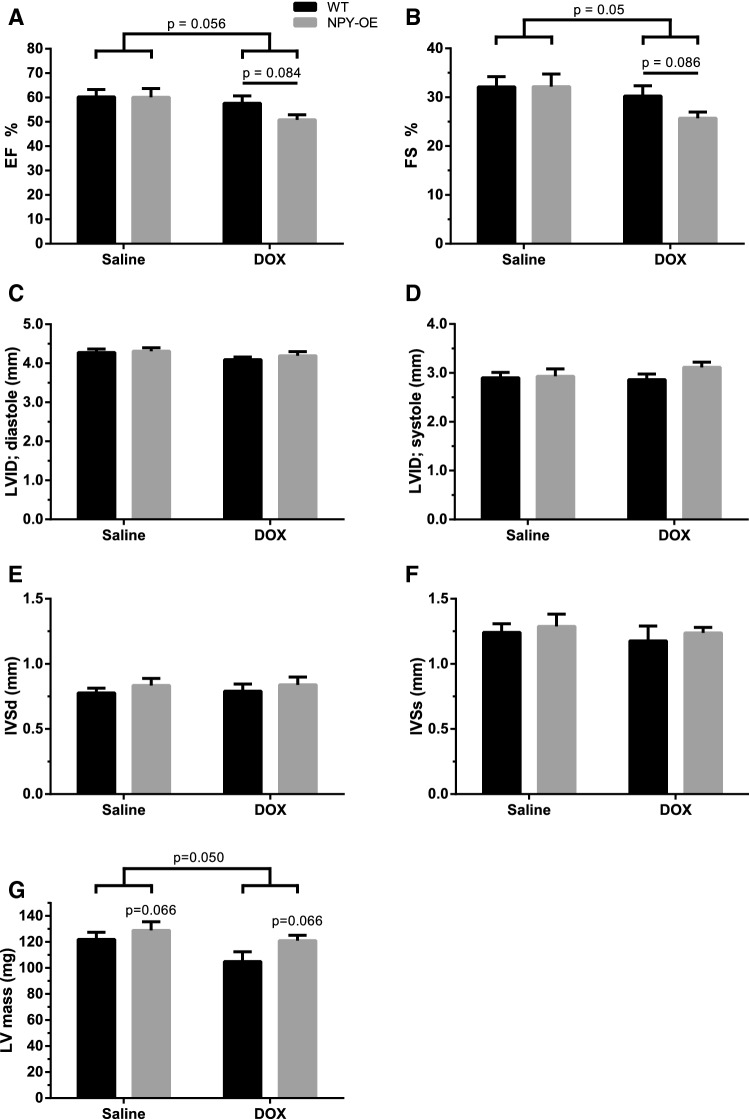
Echocardiography data in saline- and doxorubicin (DOX) -treated wild-type (WT) and NPY-OE^DβH^ (NPY-OE) mice (*n* = 7–8/group), **a** Ejection fraction (EF), **b** fractional shortening (FS), **c** left ventricle internal diameter, diastole (LVID, diastole), **d** left ventricle internal diameter, systole (LVID, systole), **e** interventricular septum, diastole (IVSd), **f** interventricular septum, systole (IVSs), **g** left ventricle mass (LV mass). Values are presented as means ± SEM. DOX vs. saline, two-way ANOVA treatment effect *p* = 0.056, *p* = 0.050; NPY-OE vs. WT, two-way ANOVA genotype effect *p* = 0.066; DOX-treated NPY-OE vs WT, *t*-test *p* = 0.084, *p* = 0.086

Echocardiography was conducted at week six. DOX treatment tended to decrease ejection fraction (NPY-OE^DβH^ DOX 50.8 ± 2.1% and WT DOX 57.6 ± 3.0% compared to NPY-OE^DβH^ saline 60.1 ± 3.6% and WT saline 60.2 ± 3.1%, *p* = 0.056) (Fig. 2a). In addition, the decrease in ejection fraction tended to be larger in NPY-OE^DβH^ mice compared to DOX-treated WT mice (*p* = 0.084) (Fig. 2a). Fractional shortening follows these tendencies of DOX treatment (NPY-OE^DβH^ DOX 25.7 ± 1.3% and WT DOX 30.2 ± 2.1% compared to NPY-OE^DβH^ saline 32.2 ± 2.6% and WT saline 32.1 ± 2.1%, *p* = 0.050) and comparing DOX-treated NPY-OE^DβH^ mice to WT mice (*p* = 0.086) (Fig. 2b), there were no differences in the left ventricle internal diameter in diastole or systole (Fig. 2c, d) or in interventricular septum in systole and diastole (Fig. 2e, f). Moreover, DOX tended to decrease the mass of the left ventricle (NPY-OE^DβH^ DOX 121.1 ± 4.1 mg and WT DOX 104.9 ± 7.5 mg compared to NPY-OE^DβH^ saline 129.0 ± 6.6 mg and WT saline 121.9 ± 5.6 mg, *p* = 0.050) and at the same time the mass of the left ventricle tended to be decreased in NPY-OE^DβH^ mice (*p* = 0.066) (Fig. 2g).

### Heart Pathology

**Fig. 3:**
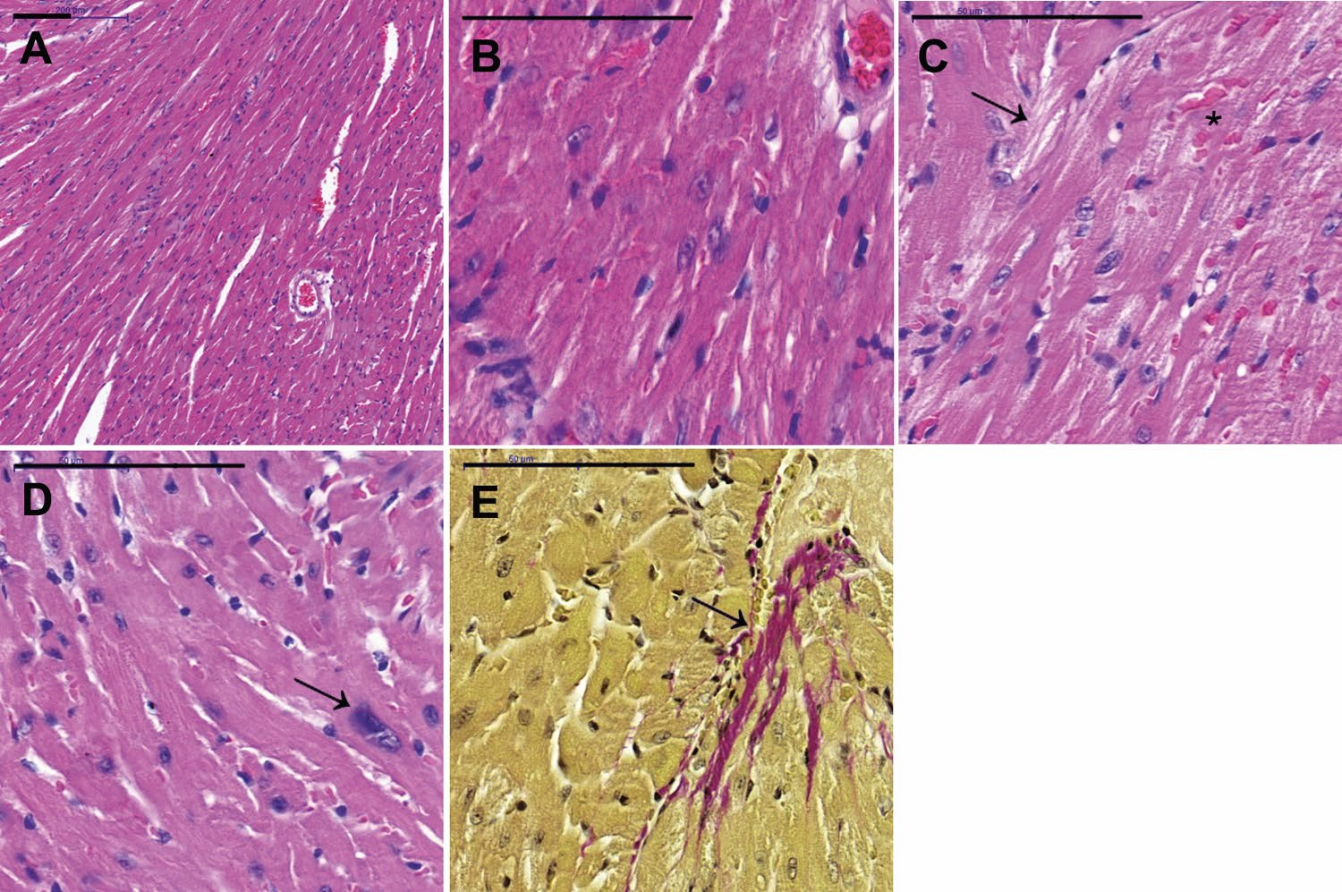
Examples of histological findings in the paraffin sections of the heart stained with hematoxylin/eosin and Van Gieson’s staining, **a** normal, saline-treated mouse; 10× zoom, **b** normal, saline-treated mouse; 40× zoom, **c**–**e** doxorubicin-treated mouse; 40× zoom. Scale bar indicates 100 µm length

In histological analysis, saline-treated hearts were histologically normal as no nuclear atypia, changes in nuclear size, fibrosis, or perivascular inflammation were seen (Fig. 3a, b). As an example of findings, in DOX-treated NPY-OE^DβH^ hearts, there was seen some cytoplasmic degeneration (Fig. 3c, arrow) and hypertrophy of the myocardial cell nuclei (Fig. 3d, arrow). In addition, some extravasated red blood cells (Fig. 3c, asterisk) and mild fibrosis were seen (Fig. 3e, arrow). In DOX-treated WT hearts, there were changes in nuclear size and hyperchromacy, as well as in cytoplasmic degeneration of myocardial cells. The histological findings were scored and the results were compared between the different treatment groups, but in statistical analysis the histological scoring failed to show any statistical significance. DOX treatment tended to decrease the weight of the heart (*p* = 0.061) and heart/tibia ratio (*p* = 0.073) compared to saline controls (Table 1). The weight of the liver or the lungs did not differ between the groups.

**Table 1:** Organ weights and tibia lengths of the saline- and doxorubicin -treated wild-type and NPY-OE^DβH^ mice

	WT saline	NPY-OE saline	WT DOX	NPY-OE DOX
Heart (mg)	148.5 ± 6.2	147.7 ± 4.1	133.6 ± 3.1	142.9 ± 5.4
Lungs (mg)	138.1 ± 8.0	144.9 ± 3.9	154.3 ± 8.8	157.0 ± 7.0
Liver (g)	1.45 ± 0.12	1.57 ± 0.09	1.47 ± 0.06	1.47 ± 0.04
Tibia (mm)	17.6 ± 0.05	17.5 ± 0.09	17.6 ± 0.04	17.6 ± 0.02
Heart/Tibia	8.39 ± 0.39	8.44 ± 0.25	7.60 ± 0.16	8.11 ± 0.31

*WT* wild-type mice, *NPY*-*OE* neuropeptide Y overexpressive mice, *saline* saline treatment, *DOX* doxorubicin treatment

Values are presented as mean ± SEM. Statistics were analyzed with two-way ANOVA (*n* = 6–8/group)

### Expression of Npy-Related Genes in the Heart

**Fig. 4:**
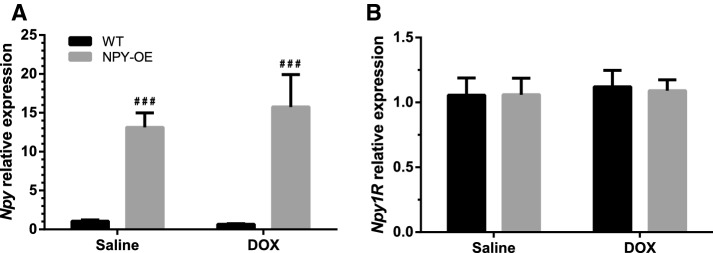
Relative mRNA expression level in the heart in saline and doxorubicin (DOX) -treated wild-type (WT) and NPY-OE^DβH^ (NPY-OE) mice (*n* = 7–8/group), **a** neuropeptide Y (*Npy*) level and **b** neuropeptide Y 1 receptor (*Npy1R*) level. Values are presented as means ± SEM, ### *p* < 0.001, NPY-OE vs. WT, two-way ANOVA genotype effect

In order to analyze the expression level of NPY-related genes in the heart, *Npy-, Npy1R-,* and *Npy5R*-relative mRNA levels were quantified with qPCR. *Npy* expression in the heart was low, but it was found to be over ten times higher in NPY-OE^DβH^ mice than in normal WT mice (Fig. 4a). DOX treatment did not alter *Npy* expression. There were no differences in neuropeptide Y 1 receptor expression levels (Fig. 4b) and the expression level of neuropeptide Y 5 receptor was under the reliable detection level by qPCR.

### Effect of Doxorubicin on Genes Modifying Calcium Cycling System

**Fig. 5:**
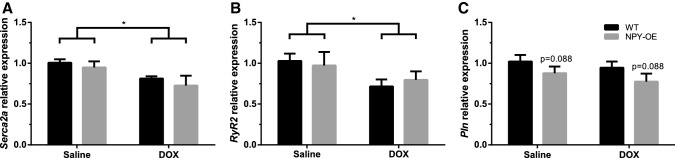
Relative mRNA expression levels of genes modifying calcium cycling system in the heart in saline- and doxorubicin (DOX) -treated wild-type (WT) and NPY-OE^DβH^ (NPY-OE) mice (*n* = 5–8/group), **a** sarcoplasmic reticulum Ca^2+^ ATPase (*Serca2a*) level, **b** ryanodine receptor (*RyR2*) level, **c** phospholamban (*Pln*) level. Values are presented as means ± SEM, * *p* < 0.05 for DOX vs. Saline, two-way ANOVA treatment effect; *p* = 0.088 for NPY-OE vs WT, two-way ANOVA genotype effect

DOX treatment resulted in lower *Serca2a* (Fig. 5a) and *RyR2* (Fig. 5b) levels compared to saline-treated mice. In *Serca2a* and *RyR2* expression, there were no differences between the NPY-OE^DβH^ and WT genotypes. In the NPY-OE^DβH^ genotype, *Pln* expression tended to be decreased compared to WT mice (Fig. 5c), but the expression was not affected by DOX treatment.

### Effect of Doxorubicin on Marker Genes of Cardiomyopathy

**Fig. 6:**
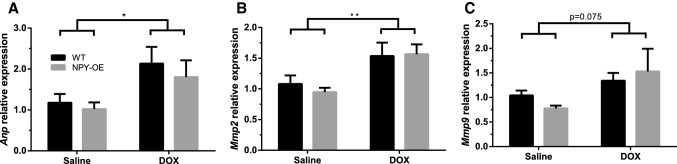
Relative mRNA expression levels of cardiomyopathy marker genes in the heart in saline- and doxorubicin (DOX) -treated wild-type (WT) and NPY-OE^DβH^ (NPY-OE) mice (*n* = 5–8/group), **a** atrial natriuretic peptide (*Anp*) level, **b** matrix metalloproteinase 2 (*Mmp2*) level, **c** matrix metalloproteinase 9 (*Mmp9*) level. Values are presented as means ± SEM, * *p* < 0.05, ** *p* < 0.01, *p* = 0.075, DOX vs. saline, two-way ANOVA treatment effect

*Anp* gene expression levels were significantly higher in DOX-treated mice compared to saline-treated mice (Fig. 6a). DOX altered also the expression of two genes involved in the degradation of proteins. *Mmp2* expression was increased (Fig. 6b) and *Mmp9* tended to be increased (Fig. 6c) by DOX. There were no differences in *Bnp* and *Mmp13* expression (Online Resource 1a, b) or in the expression levels of cytokines measured including *IL*-*1β, Tgf*-*β* ,and *Tnf*-*α* (Online Resource 1c–e). Regarding fibrosis, we saw no significant changes in myofibroblast marker *α*-*Sma* (Online Resource 1f).

### Effect of Doxorubicin on Genes Modifying Sympathetic Activity and Contractility

**Fig. 7:**
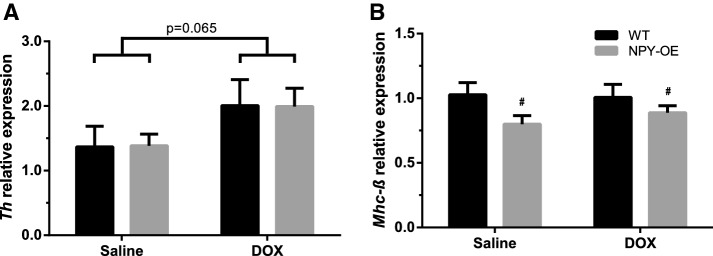
Relative mRNA expression levels of **a** tyrosine hydroxylase (*Th*), **b** β-myosin heavy chain (*Mhc*-*β*) in the hearts in saline- and doxorubicin (DOX) -treated wild-type (WT) and NPY-OE^DβH^ (NPY-OE) mice (*n* = 6–8/group). Values are presented as means ± SEM, *p* = 0.065 DOX vs. saline, two-way ANOVA treatment effect; # *p* < 0.05, NPY-OE vs. WT, two-way ANOVA genotype effect

DOX treatment tended to increase the expression of tyrosine hydroxylase, *Th* (Fig. 7a), the rate-limiting enzyme in noradrenaline synthesis, while there were no differences in beta-1 adrenergic receptor expression, *Beta1R* (Online Resource 1g). *Mhc*-*β* expression was decreased in the NPY-OE^DβH^ genotype compared to WT mice while DOX had no effect (Fig. 7b). There were no differences in *Mhc*-*α* (Online Resource 1h) or mitochondrial biogenesis regulator *Pgc*-*1α* (Online Resource 1i) expression.

## Discussion

This is the first study analyzing the role of NPY in DOX-induced cardiotoxicity. To this end, a previously developed mouse model overexpressing NPY in the sympathetic nervous system was treated with DOX using a protocol that led to a decrease in heart function and clear signs of cardiac toxicity. There were only small differences between NPY-OE^DβH^ and WT mice in their response to DOX, but these tendencies suggest that NPY overexpression increased the susceptibility to cardiotoxicity.

DOX treatment with the dosing regimen used in the study, consisting of a single intraperitoneal injection at a dose of 20 mg/kg, induced a tendency of decrease in ejection fraction suggesting impaired heart function, but did not lead to severe heart failure as indicated by e.g., unchanged lung and liver weights. However, it affected the general well-being of the animals as weight gain and fat mass gain were significantly hampered, which fits with the previous reports using different doses of DOX [40–45]. Furthermore, higher expression of the rate-limiting enzyme of noradrenaline synthesis (*Th*) suggested that cardiac SNS activity was increased in DOX-treated mice, and the result fits with previous work showing DOX to upregulate TH protein level and linking DOX-induced cardiomyopathy to increased SNS activity [3, 4, 46]. On molecular level, DOX affected several previously well-described mechanisms of DOX-induced cardiotoxicity including decreases in the expression of genes involved in intracellular calcium metabolism, *Serca2a* and *RyR2* [13, 14, 16] and increases in *Anp* and *Mmp2* levels, consistent with observations in the human heart failure [47–50]. DOX-treated mice tended to have lower left ventricular mass in echocardiography and this was supported by the heart weights. Fitting with this, increased *Mmp2* expression points to cellular level activation of degradation of structural proteins. Although heart weights or histology did not reveal myocyte hypertrophy, increased *Anp* indicates that the cellular mechanisms driving hypertrophy were recruited. The histology revealed hypertrophy of the myocardial cell nuclei, cytoplasmic degeneration, and mild fibrosis in DOX-treated animals, even though the statistical significance was lacking. However, these findings are supported by previous reports of DOX treatment leading to cardiomyocyte disorganization and myofibrillar loss [40, 43, 44, 51, 52]. Thus, the DOX administration protocol used in the current study did not induce severe heart failure during the study period, but led to significant cardiotoxicity, resulting in a decline in the general condition of the mice, a slight decline in the myocardial function and mass, and affecting several well-described components of DOX-induced cardiotoxicity.

In this study, we asked if NPY has an effect on DOX-induced cardiotoxicity. Previous studies have shown a diverse role for NPY in different types of cardiomyopathies, including an increase in NPY levels and effects on several pathological processes involved also in DOX-induced cardiotoxicity [11, 30, 53–56]. Transgenic NPY overexpression had some effects on DOX-induced toxicity. First, NPY affected body composition of DOX-treated mice. Whereas body weight and fat mass gain were decreased in DOX-treated mice of both genotypes, lean mass gain was compromised significantly only in the NPY-OE^DβH^ mice. Ejection fraction tended to be decreased in DOX-treated mice, and the effect seemed to be larger in NPY-OE^DβH^ mice. These findings suggest that the NPY-OE^DβH^ mice were more severely affected by DOX. Cardiac histology or markers of calcium handling, hypertrophy, and fibrosis were not differently affected by DOX in WT and NPY-OE^DβH^ mice. PGC-1a, marker of mitochondrial biogenesis, was previously shown to be disturbed by DOX and NPY, but was not changed in the current study [27, 33, 34]. NPY overexpression genotype caused some changes that were evident in both saline- and DOX -treated mice. LV ventricular mass tended to be increased, while genes involved in the heart contractile function, *Mhc*-*β* and *Pln,* were downregulated [26, 57, 58]. The increased mass could be contributed by the known mitogenic effect of NPY on (vascular) smooth muscle cells that was evident also in NPY-OE^DβH^ mice after endothelial damage [39]. The reduction in *Mhc*-*β* and *Pln* has been associated with improved contractility [24–26, 59, 60], which could fit with the known effects of NPY on cardiac contractility [8], but is not supported by the EF results. Thus, they may rather be a sign of compensation to changes caused by NPY overexpression that render the mice more susceptible to the DOX-induced cardiotoxicity.

DOX-induced cardiotoxicity has been associated with increased SNS activity [3, 4]. Since NPY is a co-transmitter, which is released in a more prolonged manner than noradrenaline, it is likely that NPY release is also increased in this chronic stress condition. Our study did not address this question as quantitating neurotransmitter release to target tissues is demanding and requires a special experimental setting. *Npy* expression in the heart was measured, and was not changed by DOX. Interestingly, it was upregulated in the transgenic mice. This is the only non-neuronal tissue which overexpresses NPY in NPY-OE^DβH^ mice, and it is likely that the expression represents expression in the intracardiac noradrenergic neurons rather than in cardiac myocytes [8]. However, the expression level was still very low and the SNS-derived NPY is likely responsible for any direct effects on cardiac tissue. In the transgenic mouse model used, *Npy* expression is driven under the dopamine-β-hydroxylase promoter (DβH) targeting the transgene expression to noradrenergic neurons with very little ectopic expression shown [35]. *Npy* expression is almost twice as high in noradrenergic regions of the brain and significantly higher in adrenal glands compared to WT mice leading to increased protein levels in these tissues [36]. This fairly modest increase may represent the increase in NPY during mild chronic stress or due to genetic variants, but not the high levels that have been associated with poor survival in heart failure patients [61].

NPY-OE^DβH^ mice have previously been shown to present with a metabolic syndrome -like phenotype (37), and fitting with this NPY-OE^DβH^ mice gained more weight compared with WT mice in the current experiment. The metabolic changes seem to be mediated mostly via inhibition of adrenergic tone to the metabolic tissues rather than direct effects of NPY on adipose tissue and the liver. This has been evidenced by decreased *Th* expression in the brain noradrenergic nuclei and the adrenal gland, decreased urinary adrenaline levels, and relevant changes in the target tissue beta receptor expression [36, 62]. In the current experiment, the neuronal *Th* was not studied but *Npy* had no effect on cardiac *Th* or *beta1*-receptor. These findings do not exclude the possibility that changes in the autonomic nervous system control of the heart could play a role. Furthermore, it is possible that the effects of NPY overexpression on the vascular system (including increased blood pressure) could play a role.

In summary, in our model, DOX at a relatively low dose caused significant cardiotoxicity. There were differences between NPY-OE^DβH^ and WT mice in their responses to DOX suggesting increased susceptibility to cardiotoxicity associated with NPY overexpression. This may point to therapeutic implications as suggested for NPY system in other cardiovascular diseases.

## Electronic supplementary material

Below is the link to the electronic supplementary material.

Supplementary material 1 (PDF 134 kb)
