# Supplementary material for: The Effects of Neuropeptide Y Overexpression on the Mouse Model of Doxorubicin-Induced Cardiotoxicity
Source: Cardiovasc Toxicol. 2019 Dec 6;20(3):328–38. doi: 10.1007/s12012-019-09557-2 (PMC7176599; doi:10.1007/s12012-019-09557-2)
Supplement: Supplementary file 1 — Supplementary material 1 (PDF 134 kb) [file 12012_2019_9557_MOESM1_ESM.pdf]

Cardiovascular Toxicology

The effects of neuropeptide Y overexpression on the mouse model of doxorubicin-induced cardiotoxicity

Minttu Mattila[1,2], Mirva Söderström[3], Liisa Ailanen[1], Eriika Savontaus[1,4], Mikko Savontaus[5]

[1]Research Centre for Integrative Physiology and Pharmacology, Institute of Biomedicine, University of Turku, Turku, Finland

[2]Drug Research Doctoral Programme, University of Turku, Turku, Finland

[3]Department of Pathology, Turku University Hospital and University of Turku, Turku, Finland

[4]Clinical pharmacology, Turku University Hospital, Turku, Finland

[5]Heart Centre, Turku University Hospital and University of Turku, Turku, Finland

Correspondence: Eriika Savontaus, eriika.savontaus@utu.fi

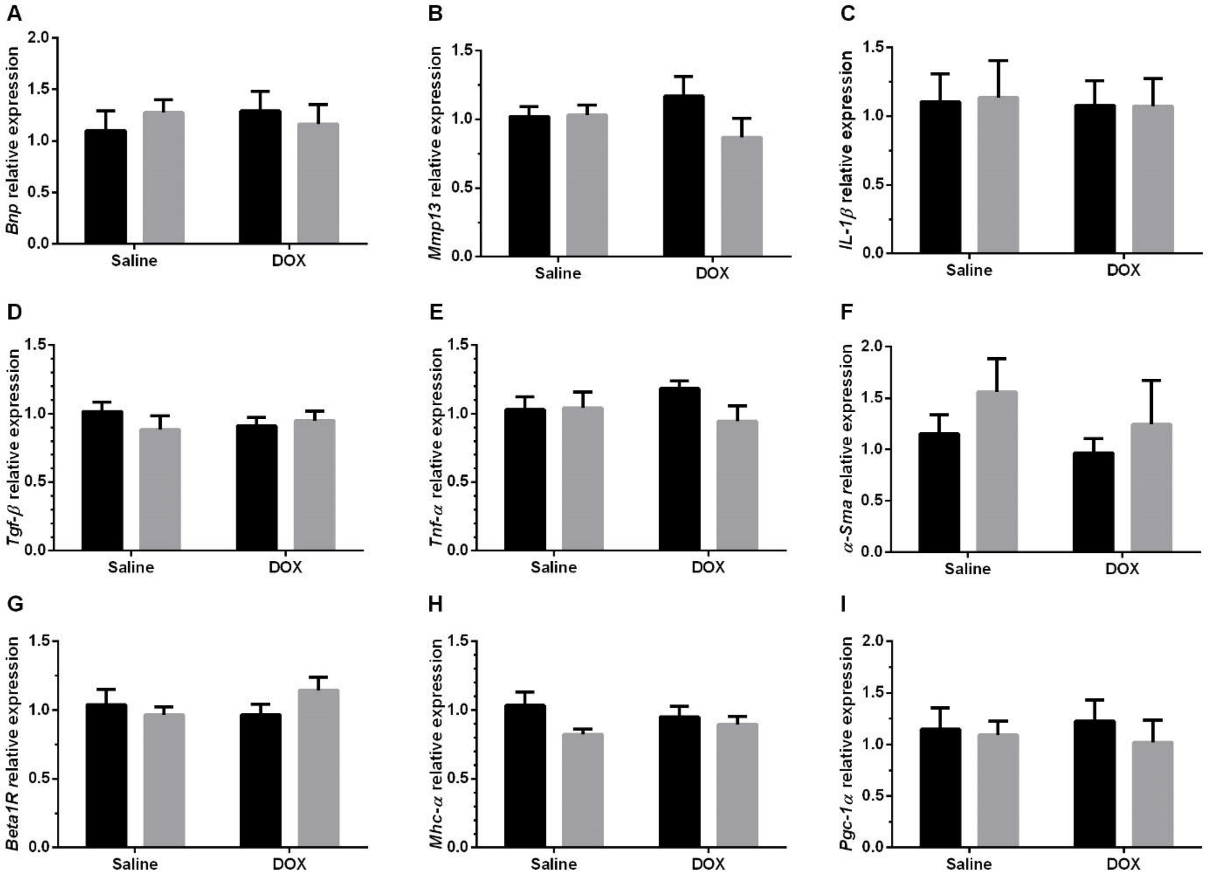

Online Resource 1 Relative mRNA expression levels in the hearts in saline and doxorubicin (DOX) -treated wild-type (WT) and NPY-OE$^{D\beta H}$ (NPY-OE) mice, a) B-type natriuretic peptide (*Bnp*) level, b) matrix metalloproteinase (*Mmp13*) level, c) interleukin-1β (*Il-1β*) level, d) transforming growth factor-β (*Tgf-β*) level, e) tumor necrosis factor-α (*Tnf-α*) level, f) α-smooth muscle actin (*α-Sma*) level, g) beta-1 adrenergic receptor (*Beta1R*) level, h) myosin heavy chain-α (*Mhc-α*) level, i) peroxisome proliferator-activated receptor-γ coactivator-1α (*Pgc-1a*) level. Values are presented as means ± SEM (n = 6-8/group)
